# Analysis of mammalian circadian clock protein complexes over a circadian cycle

**DOI:** 10.1016/j.jbc.2023.102929

**Published:** 2023-01-20

**Authors:** Xuemei Cao, Li Wang, Christopher P. Selby, Laura A. Lindsey-Boltz, Aziz Sancar

**Affiliations:** Department of Biochemistry and Biophysics, University of North Carolina School of Medicine, Chapel Hill, North Carolina, USA

**Keywords:** circadian clock, cryptochrome, PER complex, CLOCK–BMAL1 heterodimer, CCG, circadian-controlled genes, IP, immunoprecipitation, HCD MS/MS, Higher energy Collision Dissociation mass spectrometry, LC-MS/MS, liquid chromatography–tandem mass spectrometry

## Abstract

Circadian rhythmicity is maintained by a set of core clock proteins including the transcriptional activators CLOCK and BMAL1, and the repressors PER (PER1, PER2, and PER3), CRY (CRY1 and CRY2), and CK1δ. In mice, peak expression of the repressors in the early morning reduces CLOCK- and BMAL1-mediated transcription/translation of the repressors themselves. By late afternoon the repressors are largely depleted by degradation, and thereby their expression is reactivated in a cycle repeated every 24 h. Studies have characterized a variety of possible protein interactions and complexes associated with the function of this transcription–translation feedback loop. Our prior investigation suggested there were two circadian complexes responsible for rhythmicity, one containing CLOCK–BMAL and the other containing PER2, CRY1, and CK1δ. In this investigation, we acquired data from glycerol gradient centrifugation and gel filtration chromatography of mouse liver extracts obtained at different circadian times to further characterize circadian complexes. In addition, anti-PER2 and anti-CRY1 immunoprecipitates obtained from the same extracts were analyzed by liquid chromatography–tandem mass spectrometry to identify components of circadian complexes. Our results confirm the presence of discrete CLOCK–BMAL1 and PER–CRY–CK1δ complexes at the different circadian time points, provide masses of 255 and 707 kDa, respectively, for these complexes, and indicate that these complexes are composed principally of the core circadian proteins.

The mammalian circadian clock controls many aspects of physiology and behavior, allowing organisms to live in sync with the daily light/dark cycle. The manifestations of the clock follow from cyclical expression of circadian-controlled genes (CCGs) in cells throughout the body (1, 2, 3, 4, 5). Expression of CCGs is controlled *via* E-box elements in their promoters, to which the CLOCK–BMAL1 transcriptional activator complex binds. Repressors of CLOCK–BMAL1 include the PER and CRY proteins (PER1, PER2, PER3, and CRY1, CRY2), and CK1δ. The repressors themselves are CCGs, and in cells throughout the body, there is a daily rhythm beginning in the morning with CCG activation by CLOCK–BMAL1, and expression of PERs, CRYs, and CK1δ. The latter are then translocated to the nucleus where they repress CLOCK–BMAL1-mediated expression. Following degradation of the repressors, gene activation by CLOCK–BMAL1 begins again (6, 7, 8, 9, 10, 11). Besides this main transcription–translation feedback loop, the clock-controlled REV–ERBα and REV–ERBβ regulators of BMAL1 and CRY expression provide secondary “loops” that contribute in stabilizing the rhythmicity (10).

Interestingly, chromation immunoprecipitation, immunoprecipitation (IP), and gene expression–based studies have indicated that repression of CLOCK–BMAL1-activated transcription occurs by two mechanisms: CRY alone apparently binds to the activation complex and directly represses (“blocking-type repression”), or PER–CK1δ, in a CRY-dependent manner, removes CLOCK–BMAL from the E-box (“dissociation-type repression”) (6, 11, 12, 13, 14, 15, 16). In dissociation-type repression, CRY apparently aids in bringing PER–CK1δ to the CLOCK–BMAL1 activator; CK1δ then phosphorylates CLOCK so as to destabilize its association with DNA.

Biochemists have long attempted to characterize circadian protein complexes responsible for the rhythmic activity of the core circadian clock proteins. For the activation phase, CLOCK and BMAL1 were found to bind E-boxes as a heterodimer (17, 18, 19). In investigations including repressive phase proteins, CLOCK, mPER2, and mCRY2 were reported to form complexes with a wide range of sizes (200–5000 kDa) based upon gel filtration data (20). Since then, additional studies using gel filtration, coimmunoprecipitation, and blue native-agarose gel electrophoresis have discovered a wide range of components principally in PER-containing complexes, including circadian clock and non–circadian clock proteins and RNAs, capable of assembling in a megadalton-sized complex with PERs (21, 22, 23, 24, 25, 26, 27). However, several factors reported as components of PER complexes were found to be present in substoichiometric amounts (26), and it is unclear whether they were present as integral clock components, partners involved in noncanonical clock or nonclock functions (28, 29, 30, 31, 32), or simply as components comingled in concentrated complex mixtures.

In a more recent investigation, using glycerol gradient sedimentation of nuclear extracts made from mouse liver, we identified two circadian complexes, one containing CLOCK and BMAL1, and a second, larger complex containing PER2, CRY1, and CK1δ (15). As in prior studies, protein standards were included in the gradients to estimate the mass of the complexes. However, since gradients separate based upon sedimentation coefficient (S), not mass, such estimates may not be accurate. To further probe the structure–function relationships in circadian rhythmicity, we used both glycerol gradient centrifugation and gel filtration chromatography to obtain values for S and Stokes radius (R) for circadian proteins and complexes. These values, which describe the size and shape of globular and nonglobular proteins/complexes, can be used in combination as described previously to calculate the mass of proteins/complexes (33, 34). We also performed IPs using both PER2 and CRY1 antibodies followed by liquid chromatography–tandem mass spectrometry (LC-MS/MS) analysis to identify components of circadian complexes. Our results confirm the identification of circadian activator (CLOCK–BMAL1) and repressor (PER–CRY–CK1δ) complexes and assign their masses as 255 and 707 kDa, respectively. Our results also show that these complexes consist principally of circadian protein and provide a structural basis for circadian protein functioning.

## Results

### Glycerol gradient analysis of circadian proteins

**Figure 1:**
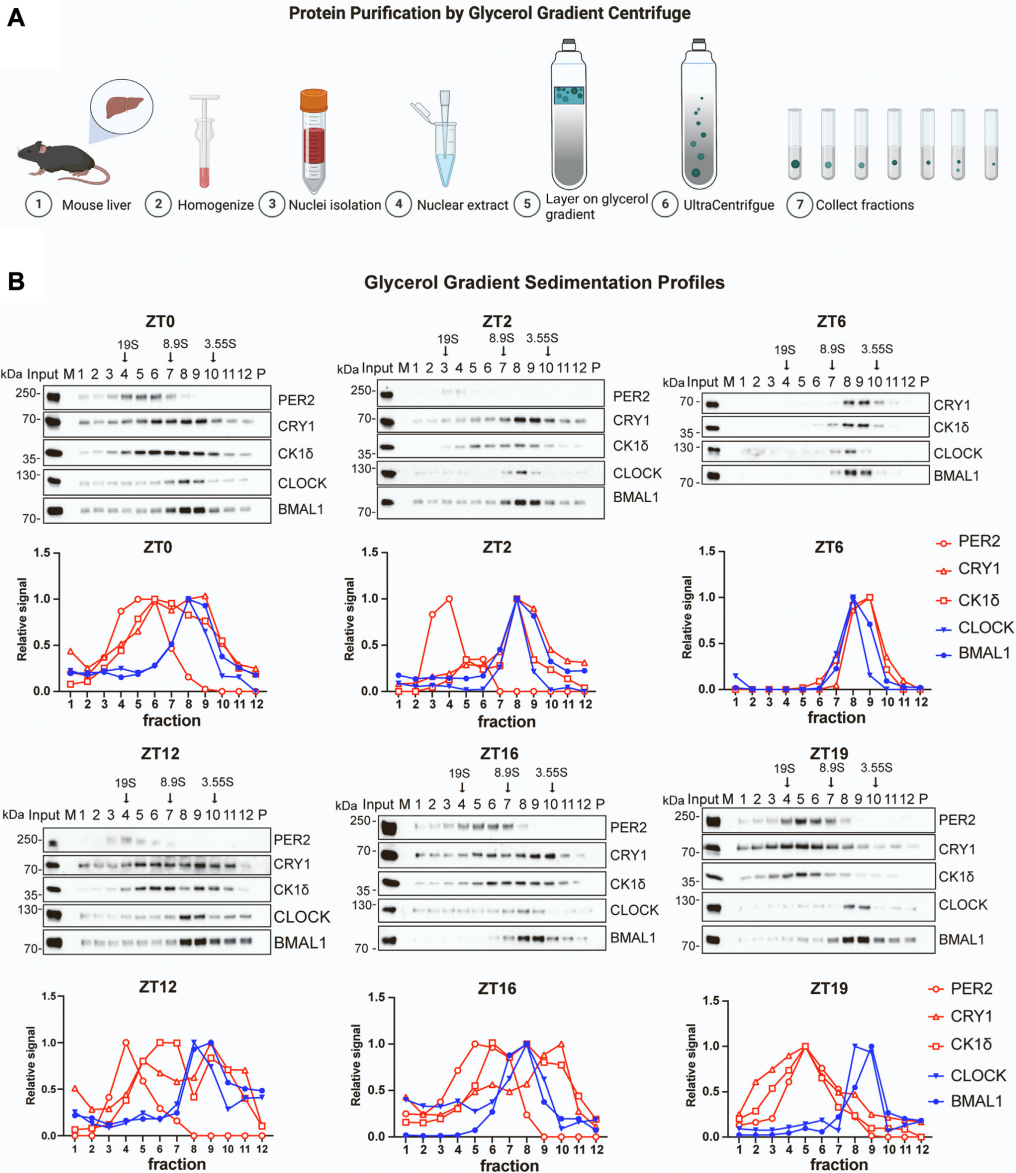
**Analysis of nuclear circadian complexes by glycerol gradient centrifugation.***A*, method: Mouse nuclear extract and reference proteins were mixed, layered on a 10 to 30% glycerol gradient, and centrifuged, and then fractions were collected from the bottom for analysis by SDS gel electrophoresis followed by Western blot (Fig. 1*B*) and Coomassie blue staining (Fig. S1). Reference proteins included bovine thyroglobulin (669 kDa, 19 S), sweet potato beta-amylase (222 kDa, 8.9 S), and chicken ovalbumin (43 kDa, 3.55 S). *B*, sedimentation profiles for PER2, CRY1, CK1δ, CLOCK, and BMAL1 determined for extracts of mice harvested at ZT0, ZT2, ZT6, ZT12, ZT16, and ZT19. For each ZT, the Western blot is above a graph showing quantitative values for band intensity of each protein relative to each protein’s peak intensity, given a value of 1. Arrows indicate positions of the peak fraction of each reference protein as determined from Coomassie blue–stained gels with the same samples (Fig. S1). In the blots, “P” stands for pellet; this sample was obtained by washing the emptied gradient tube with 240 ul buffer; the purpose of analyzing this sample was to detect and characterize any insoluble material that might pellet during centrifugation. No proteins were detected in the pellets. Three percent of each extract was loaded directly to the gels to indicate “Input.” Ten percent of each fraction was loaded to the gels. Three repeats were done for each ZT, and essentially identical data were obtained at each ZT. Representative images are shown.

To examine circadian protein interactions, nuclear extracts from mouse livers harvested at six time points (ZT0, ZT2, ZT6, ZT12, ZT16, and ZT19) were mixed with protein markers and subjected to glycerol gradient centrifugation, and fractions were analyzed for specific proteins by Western blotting, as outlined in Figure 1*A* and S1. CRY1 and PER2 served as representative of the CRYs and PERs, respectively, as they are the most reliably measured. In a prior study using this approach, we found that at ZT19, when PER and CRY are at peak levels, there are two separate complexes: CLOCK–BMAL, which migrated at a position corresponding to approximately 200 kDa, and CRY, PER, and CK1δ, which comigrated at approximately 550 kDa. These values were based upon the molecular weights of the protein markers (15) and are estimates since migration is based not on mass but sedimentation coefficient. A goal of the current investigation is to examine the molecular weights of the complexes more directly, and toward this end we will consider the sedimentation coefficients (S) of the circadian complexes in combination with their Stokes radii as discussed below.

Sedimentation profiles of the circadian proteins and protein standards obtained at different circadian times are shown in Figures 1*B* and S1. The profiles in Figure 1*B* show separate elution of CLOCK–BMAL and PER–CRY–CK1δ, especially at ZT19 when PER levels are highest, as reported previously (15). The mobility of CLOCK–BMAL at approximately 7.9S does not change appreciably as a function of circadian time, and the two proteins largely overlap, although there is a trend for more BMAL in lower S value fractions perhaps owing to limiting amounts of CLOCK for complex formation, and CLOCK-free BMAL migrating as a monomer or associated with smaller protein(s). The relatively constant profiles of these two proteins across time points is consistent with the fact that CLOCK and BMAL1 protein levels are not known to vary considerably with circadian time.

An apparent PER–CRY–CK1δ complex migrates at approximately 15.6S at most circadian times, including ZT19, when expression of the repressors peaks. At the other extreme, ZT6, when PER is undetected, all CRY1 and CK1 are apparently monomeric. When PER is at intermediate levels, CRY and CK1δ migrate with PER2, and also in lower S-value fractions. Thus, during much of the day, PER appears to be limiting for formation of PER–CRY–CK1δ complexes.

### Gel filtration analysis of circadian proteins

**Figure 2:**
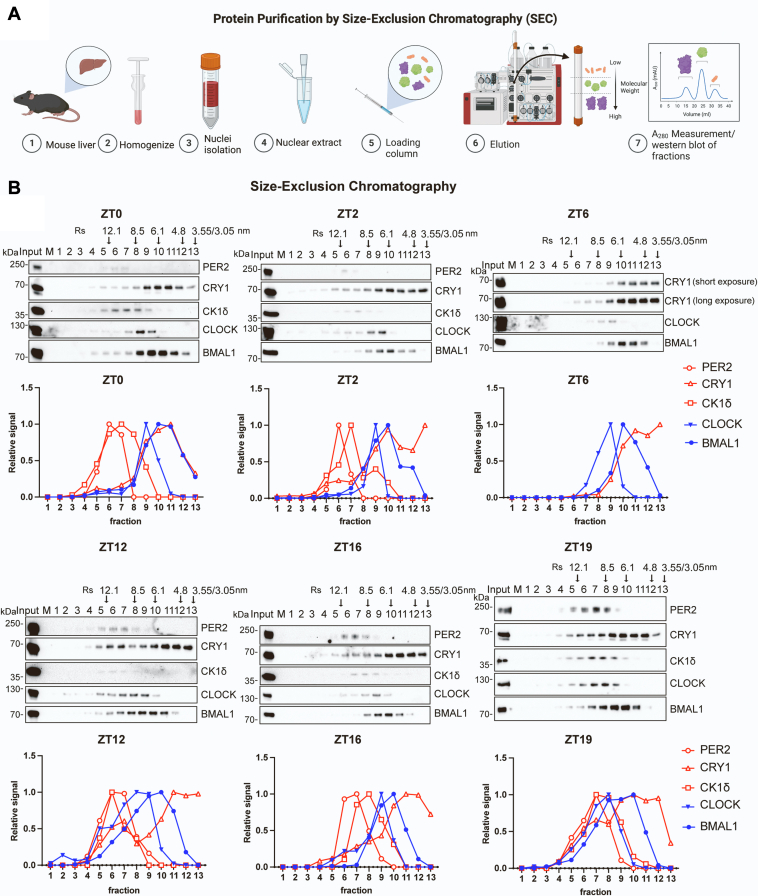
**Analysis of nuclear circadian complexes by gel filtration chromatography.***A*, method: Extracts were prepared as done for glycerol gradient analysis. Proteins in 50 to 90 ul of extract were separated using a Superose 6 Increase 10/300GL column. The column was standardized as shown in Fig. S2 using ovalbumin (45 kDa, Rs 4.8 nm), bovine serum albumin (66.5 kDa, Rs 3.55 nm), rabbit muscle aldolase (160 kDa, Rs 4.8 nm), horse spleen ferritin (440 kDa, Rs 6.1 nm), bovine thyroglobulin (669 kDa, 8.5 nm), IgM (990 kDa,12.1 nm), and Dextran (marker for void volume). *B*, elution profiles for PER2, CRY1, CK1δ, CLOCK, and BMAL1 determined for extracts of mice harvested at ZT0, ZT2, ZT6, ZT12, ZT16, and ZT19. For each ZT, the Western blot is above the graph showing quantitative values for band intensity of each protein relative to each protein’s peak intensity, given a value of 1. *Arrows* indicate positions of the peak elution fraction for each reference protein. Chromatographic analysis was done at least twice for each ZT, and representative images are shown.

To complement the above effort and to obtain more precise estimates of complex molecular weights, we examined circadian protein complexes by gel filtration chromatography using the approach shown in Figure 2*A*. Nuclear extracts from mouse livers harvested at different circadian times were prepared as the starting material. In this approach, rather than mixing the standards with the samples, the column was standardized (Fig. S2).

The elution profiles in Figure 2*B* show that as with the gradients, the sizing column separated the proteins into two separate, apparent complexes. CLOCK–BMAL1 exhibits a Stokes radius (R) of approximately 7.7 nm, and PER–CRY–CK1δ, R = 10.79 nm. Trends seen with the gradients were also seen in the gel filtration results. For one, BMAL was largely present in CLOCK-containing fractions, and it was also present in smaller R fractions, again indicating that CLOCK is a limiting component for complex formation. Regarding the PER–CRY–CK1δ complex, PER is again seen as the limiting factor since CRY and CK1δ are present at increasing amounts in lower-R-value fractions at the circadian times when the level of PER is reduced. Notably, in the apparent absence of detectable PER at ZT6, CRY migrates as an apparent monomer with a radius of 5.4 nm.

### Sizes of circadian complexes

Based on the gradient and gel filtration results, the molecular weights of the circadian complexes were calculated as described in Methods using values indicated in Table 1. We find the molecular weight of the PER–CRY–CK1δ complex is about 707 kDa, which is consistent with multimers of PER1, PER2, PER3, CRY1, CRY2, CK1δ, and Ck1ε, components that we find present in this complex (see below). It is unclear whether there is a single homogeneous repressor complex or if there are multiple repressors that vary in composition of PERs and CRYs. We find that CLOCK–BMAL1 is approximately 255 kDa, which is consistent with a CLOCK–BMAL1 heterodimer, although somewhat larger than the calculated heterodimer size. This discrepancy may be partly due to posttranslational modifications. The observed mobility of CLOCK-free BMAL1 indicates that its behavior may be influenced by interactions with itself as a dimer, or with BMAL2, NPAS2, or CRY1.

**Table 1:** Molecular weights of the circadian proteins and complexes

Complex	Calculation	Mass	Components
PER–CRY–CK1	4205∗15.6∗10.79 4205∗7.9∗7.7	707 kDa	PER1-3, CRY1-2, CK1 CLOCK–BMAL1
CLOCK/BMAL1		255 kDa	
PER1		136 kDa	
PER2		137 kDa	
PER3		132 kDa	
CRY1		66 kDa	
CRY2		67 kDa	
CK1δ		44 kDa	
CK1ε		43 kDa	
CLOCK		96 kDa	
BMAL1		69 kDa	

Molecular weights of the PERCRY–CK1 and CLOCK–BMAL1complexes were calculated (as described in Methods) as the product of 4205 x Stokes radius x sedimentation coefficient. Components of the PER–CRY–CK1 repressor complex are shown; however, it is not known if a single repressor complex exists or if there is a population of repressors, each with different amounts/stoichiometries of the repressor proteins. Below the two complexes, individual clock proteins are listed with their masses, calculated from their sequence. The experimentally derived mass of the CLOCK–BMAL1 complex is consistent with a heterodimer, although somewhat larger than the calculated value. This discrepancy may be partly due to protein modifications present in the complex. The three experiments gave the same values for the complex masses since the peak fraction for the complex was the same in relation to the standards in each experiment. As a way to assess potential variability in the results, we calculated complex molecular weights assuming the peak elution fraction was one fraction earlier or later. In the case of gel filtration, if peak elution had been one fraction earlier or later, then the 707-kDa complex would have been 875 or 589 kDa, respectively, and the 255-kDa complex would have been 336 or 171 kDa. In the case of glycerol gradients, if peak elution had been one fraction earlier or later, then the 707-kDa complex would have been 793 or 557 kDa, respectively, and the 255-kDa complex would have been 282 or 215 kDa.

### Components of circadian complexes

**Figure 3:**
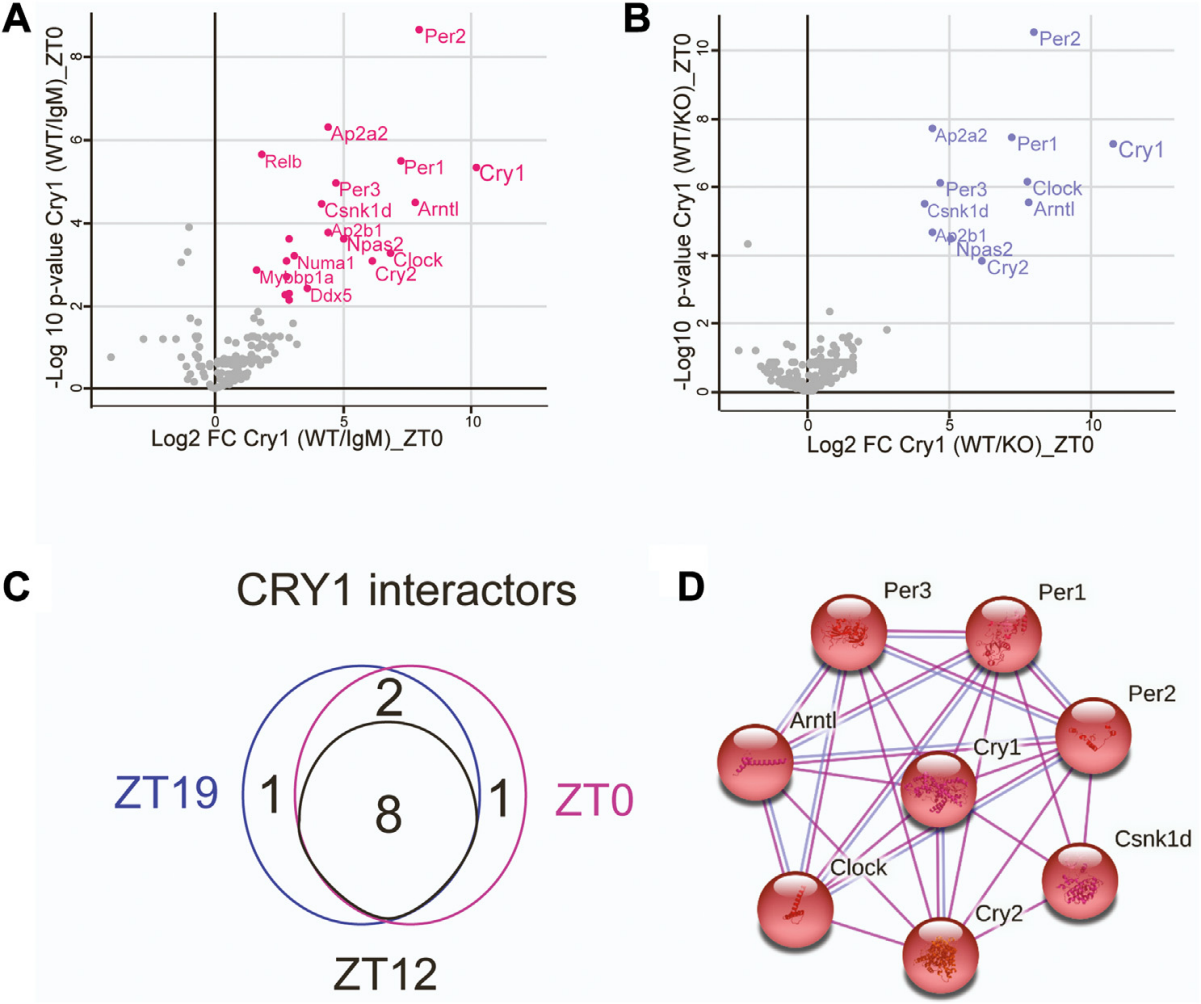
**CRY1 interacting proteins.** Nuclear extracts prepared from mouse liver at ZT0, ZT12, and ZT19 were immunoprecipitated with anti-CRY1 antibodies. Two negative controls were used: IgM antibody immunoprecipitation (IP) of wildtype liver extract and anti-CRY2 immunoprecipitation of *Cry1/2*^*−/−*^ knockout (KO) mouse liver extract. *A*, Volcano plot showing proteins detected by IP, using IgM as the negative control. The ratio, or fold change (FC) of binding to antibody *versus* IgM (WT/IgM) is plotted as Log2 FC CRY1 (WT/IgM) on the *x*-axis *versus* the -log10 *p*-value for the fold change (*y*-axis). Proteins showing log2 FC > 1 and -log10 *p* value >2 are defined as specific interactors (*pink dots*) of statistical significance. *B*, volcano plot showing proteins detected by IP, using *Cry1/2*^−/−^ double KO mice as a negative control. Plotting was analogous to (A), except using ratios of WT/KO, and specific interactors of significance are in blue. *C*, Venn diagram showing common CRY1 interacting proteins identified at ZT0, ZT12, and ZT19. *D*, interactive network of the eight common CRY interacting proteins, generated by STRING.

Our results with CLOCK–BMAL1 are consistent with the finding that they activate transcription by binding to E-boxes as a heterodimer (17, 18, 19). To further assess the composition of the repressor complex(s), we immunoprecipitated nuclear extracts with either anti-CRY1 or anti-PER2 antibodies and identified bound proteins by LC-MS/MS (Tables S1 and S2). For CRY1 IPs we used extracts prepared at ZT0, ZT12, and ZT19 (Fig. S3). Negative controls for precipitating wildtype extract with CRY1 antibody included using IgM instead of the CRY1 (IgM) antibody, and using extract from *Cry1/2*^−/−^ knockout mice instead of wildtype. The data are presented in the two volcano plots in Figure 3, *A* and *B*. The dots plotted represent the proteins that were detected, which are plotted as the ratio of the amount bound in test *versus* control group (*x*-axis) as a function of the significance of the ratio (*y*-axis). (A) shows data obtained using the IgM negative control, and (B) shows data obtained using the *Cry1/2*^−/−^knockout extract as negative control. Colored dots identify the significant interactors for this experiment, which analyzed extracts from mice harvested at ZT0. More potential interactors (26) were observed when IgM was used as the control. When the double knockouts were used as a control, the 11 interactors identified were mostly core clock proteins (Fig. 3*B*), suggesting that use of the IgM control introduced artifactual interactions. For the knockout control dataset, comparing the filtered protein lists for the different circadian times and negative controls, only eight proteins overlapped, all of which were core clock proteins (Fig. 3*C*). Figure 3*D* shows the interactive network of these eight proteins as generated by STRING. These results coupled with the above findings support the existence of a circadian repressor complex that is composed of known, circadian repressor proteins PER, CRY, and CK1δ. The function of the repressor implies the presence of PER, CRY, and CK1δ. However, the analysis also identifies CLOCK and BMAL as interactors. While these components could be bona fide components of the repressor complex, because they did not comigrate substantially with the repressor (Figs. 1 and 2), it seems more likely that they were identified due to their binding to CRY1 separately from the repressor complex, or perhaps they bind in a limited manner during clock function.

**Figure 4:**
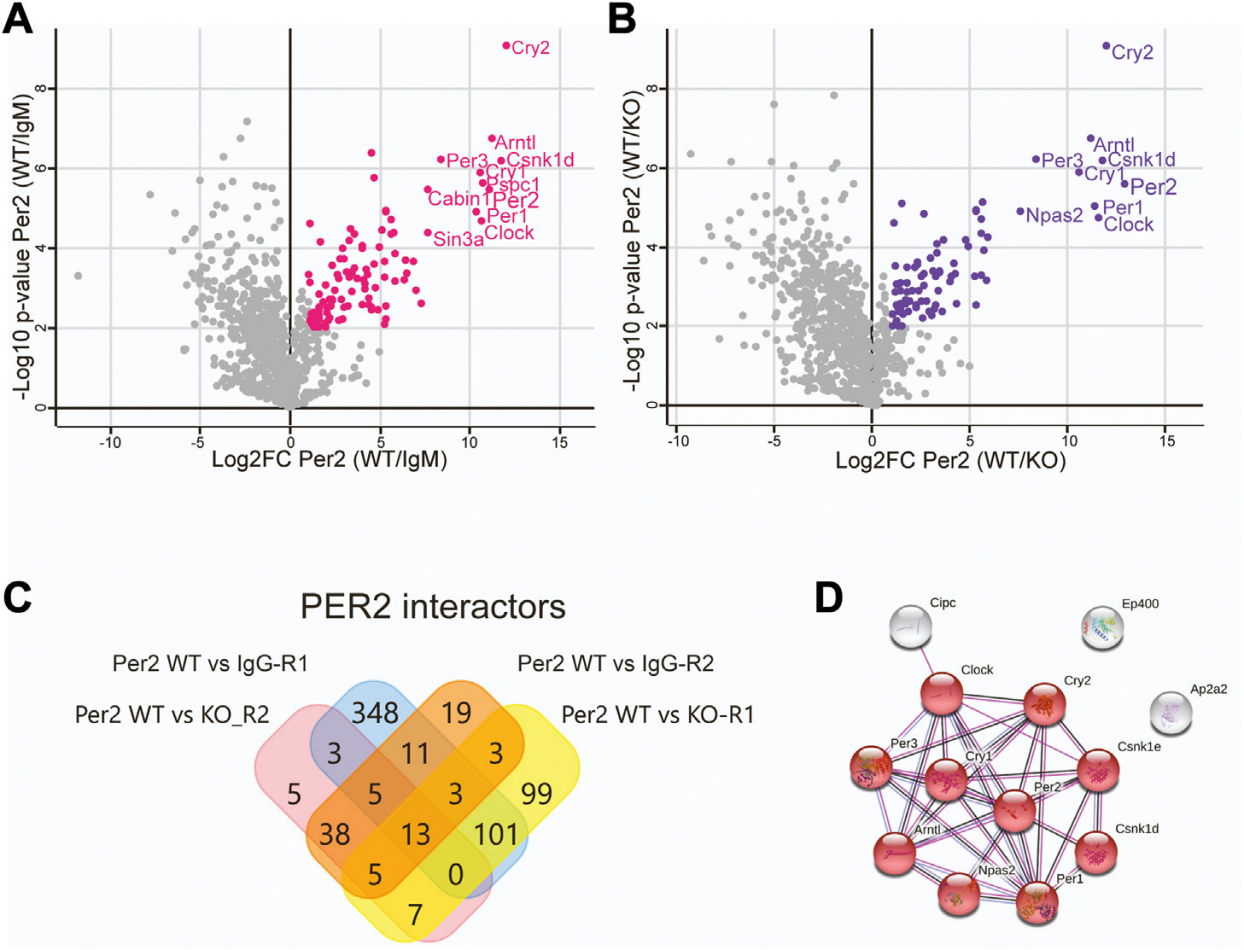
**PER2 interacting proteins.***A*, nuclear extracts prepared from mouse liver at ZT19 were immunoprecipitated with anti-PER2 antibodies. The volcano plot was prepared as in Figure 3*A*. *B*, volcano plot comparing binding ratios as a function of *p* values for proteins detected in wildtype *versus Per1/2*^*−/−*^ mouse liver nuclear extracts. *C*, Venn diagram showing overlap of significant PER interacting proteins between the two approaches (PER2 WT *versus* IgG, PER2 WT *versus* KO) and the two repeats of each approach (R1, R2). *D*, interactive network of the 13 common interacting proteins identified in (*C*), generated by STRING.

PER immunopurification was done using nuclear extracts from mice sacrificed at ZT19 (Fig. S4). Negative controls included IgM antibody and extract from *Per1/2* double knockout mice. The LC-MS/MS analysis (Table S2) identified 107 significant interactors using IgG as the negative control and 86 significant interactors using double knockouts as the negative control (Fig. 4, *A* and *B*). The Venn diagram in Figure 4*C* shows that only 13 interactors were consistently observed among the different repeats and control groups. An interactive (STRING) diagram in Figure 4*D* shows that these interactors are principally core clock proteins and include the same proteins found to interact with CRY1. These results showing limited interactions indicate that the circadian repressor complex is essentially limited to core circadian proteins. As noted above, it is not clear whether proteins including CLOCK, BMAL, and NPAS2 are components of the repressor complex; CLOCK and BMAL1 did not comigrate with the repressors (Figs. 1 and 2), and these proteins may have bound to PER2 separately from the repressor complex.

## Discussion

This study confirms and extends our prior identification and characterization of two complexes comprising circadian proteins. This study clarifies the molecular weights of these complexes as 707 (PER–CRY–CK1δ complex) and 255 kDa (CLOCK–BMAL) and shows that the composition of these complexes is limited to circadian proteins. The limiting component in these complexes appears to be PER in the repressor complex and CLOCK in the activator complex. The proteins in stoichiometric excess (CRY, CK1δ, BMAL) comigrated with their partners and also eluted in fractions of smaller size, with a trailing profile or a secondary peak or a combination of these. The trailing and secondary peaks of CRY and CK1δ tended to become more prominent as the level of PER (and CRY) decreased to a nadir at ZT6. Consistent with this observation is the finding that PER2 and CRY1 bind with a 1:1 stoichiometry (35), and following the peak expression of PER2, CRY1 becomes as much as 5- to 10-fold more abundant than PER2 (36). The secondary peak of CRY was in some cases broad and may reflect binding to free BMAL, a CRY1 dimer or CRY1-CRY2 heterodimer, or monomeric CRY1. Secondary peaks of CK1δ could arise by interaction with noncircadian proteins. Proteins present in trailing fractions and secondary peaks must contain some lower-molecular-weight subcomplexes containing PER and CLOCK and/or BMAL1, as well as CRY and CLOCK and/or BMAL1, since interactions between the repressor and activator clock proteins were detected by IP/LC-MS/molecular dynamics, and importantly, no stable “mega complex” of CLOCK–BMAL1 and PER–CRY–CK1δ was observed using gradients or gel filtration. During “blocking repression,” CRY binds to CLOCK–BMAL on DNA when the level of PER is low. We saw no clear peaks indicative of CRY–CLOCK–BMAL1 complexes, which were likely pelleted while bound to the genomic DNA during nuclear extract preparation.

The approaches selected for this study were intended to provide more certainty on a topic in which more clarity is needed (20, 21, 26, 27). The dual sedimentation coefficient/Stokes radius measurements provide complementary information and a check on reproducibility, and values obtained for these hydrodynamic parameters can be used to reliably determine protein mass. In addition, in assessing protein components of the circadian complexes, a satisfying outcome of the IP/LC-MS/MS experiments was the finding of a rather discrete overlapping set of mainly circadian proteins that bound to CRY1 and to PER2. Clearly, this observation would not have been made without the use of two negative controls, the sham immunoglobulin, and the knockout mice. Using the negative controls individually yielded numerous false binding partners, which were not observed with the complementary control or experimental repeat. In addition, in our experiments, we found that, to perform gel filtration, high-speed centrifugation of extracts was needed to ensure proper column loading. High-speed centrifugation also reduced the number of interacting partners identified by coimmunoprecipitation. Thus we find that considerable care is needed to avoid artefactual protein–protein interactions in studies of this nature.

Reported findings at variance with those described here have utilized different methods for separating complexes and different antibodies for purification. Previous studies calculated complex sizes based upon gel filtration alone or native gel mobility, both of which have limitations. Previous studies have also identified interacting partners using FLAG- or His-tagged circadian clock proteins, without complementary negative controls needed to avoid identification of nontarget proteins. A report of an approximately 1.9-mDa stable complex composed of activator and repressor circadian proteins (26) included many other factors, which were presumably filtered out in our analysis by the dual negative controls. Thus, we believe that many interacting partners reported for PER and CRY are “fellow travelers rather than conjugal partners” (37), because they are not reproducible or consistently observed by different methods.

**Figure 5:**
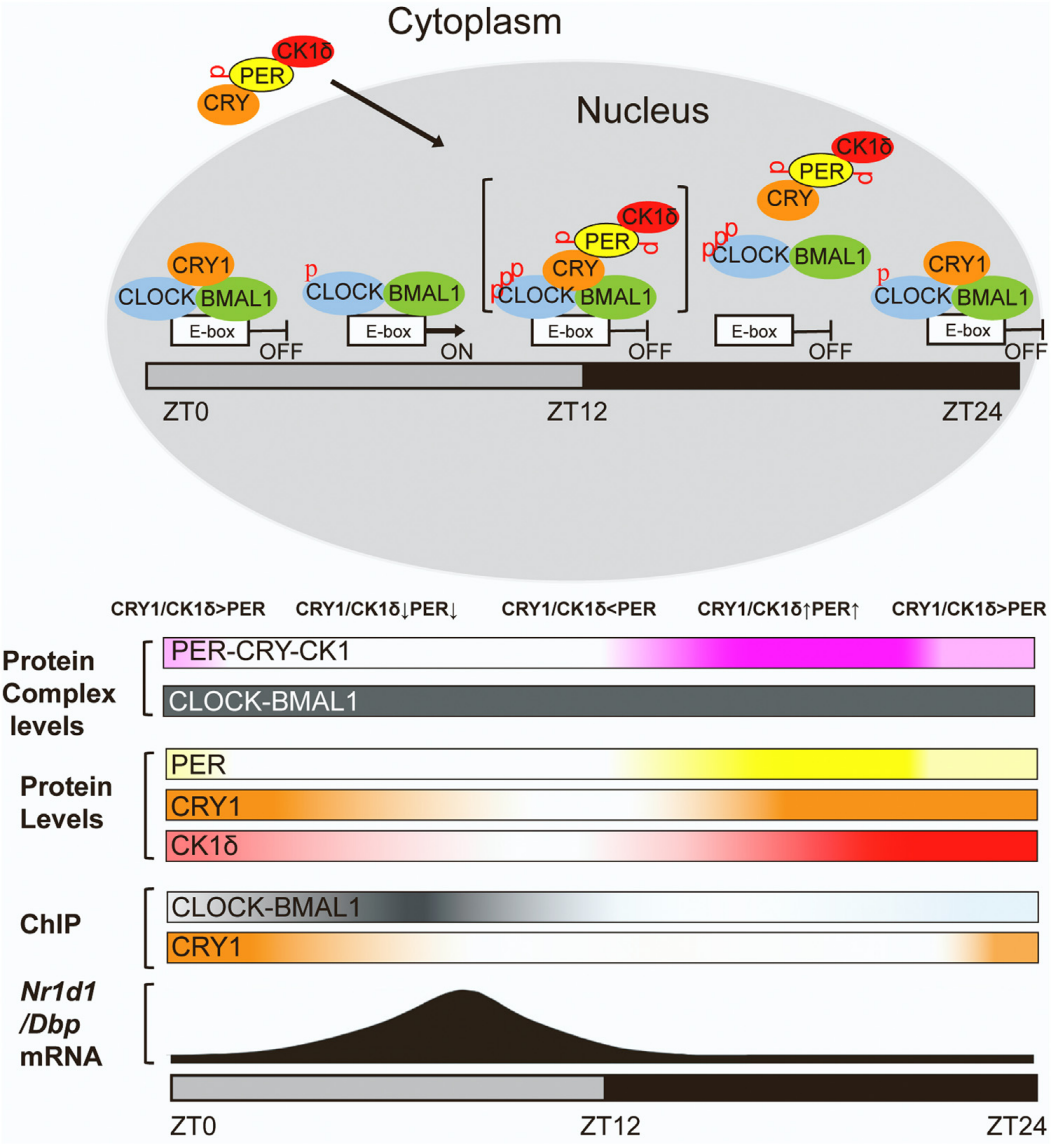
**Model showing functional and structural aspects of the core circadian clock.** The model shows cartoon images of circadian proteins and complexes in the cytosol and nucleus across a 24-h day (*top*). Below, also across a 24-h day, are shown protein and protein complex levels, binding of CLOCK–BMAL to an E-box as detected by ChIP, and RNA expression from the E-box-controlled *Nr1d1* and *Dbp* genes. Top and *bottom* images show PER in yellow, CRY in orange, CK1δ in red, the CLOCK–BMAL1 complex in gray, and the PER–CRY–CK1δ complex in *pink*. At ZT24/ZT0, PER levels are low and CRY is high, and monomeric CRY binds to CLOCK–BMAL1 complexes on DNA and inhibits transcription of *Nr1d1* and *Dbp* genes by blocking-type repression. As CRY is degraded, CLOCK–BMAL1-mediated transcription of *Nr1d1* and *Dbp* (and *Pers*, *Crys*, and *Csnk1d*) increases. Later, by lights off (ZT12), the level of repressor complex is sufficiently high to suppress transcription by transiently binding CLOCK–BMAL1 (the transient interaction is indicated by brackets), phosphorylating CLOCK, and thereby destabilizing CLOCK and removing CLOCK–BMAL1 complexes from E-boxes (displacement-type repression). Through the dark hours and up to around ZT24, as PER is more rapidly degraded than CRY, dissociation-type repression of CLOCK–BMAL1 is replaced by blocking-type repression by CRY due to the binding of CRY alone to CLOCK/BMAL1. Modified from (6, 12, 13, 15).

The structural characterizations reported here are consistent with the functions of these circadian proteins. The model in Figure 5 shows, from top to bottom, the structure–function relationships for the circadian complexes, expression levels of the circadian proteins and complexes in the nucleus, the extent of CLOCK and CRY1 binding to an E-box measured by chromation immunoprecipitation experiments, and expression of the E-box-controlled genes *Nr1d1* and *Dbp* over the course of a circadian cycle. As the day begins (ZT0), PER levels become depleted, and sufficient levels of monomeric CRY remain, allowing CRY to bind to CLOCK–BMAL1 on DNA and inhibit CLOCK–BMAL1-mediated gene activation at E-boxes in a phase of “blocking-type repression.” During the day, CRY is further degraded, and expression of E-box-controlled genes is derepressed. Eventually, PER, CRY, and CK1δ levels increase, they form a complex, and translocate to the nucleus. By approximately lights out, ZT12, the repressor complex transiently binds to CLOCK–BMAL1 and phosphorylates CLOCK. Since the binding is transient, no stable (CLOCK–BMAL1)–(PER–CRY–CK1δ) was detected in our experiments, and the transient nature of the complex is indicated with brackets. The destabilized CLOCK then dissociates from the E-box in this phase of “dissociation-type repression,” as described previously (14, 16). Later, before lights on, although PER levels are substantially diminished, CRY degradation is slower and CRY continues “blocking-type repression” of any CLOCK–BMAL1 remaining bound to E-boxes at ZT24/ZT0.

In “displacement-type repression,” the repressor complex transiently binds to CLOCK–BMAL1 on DNA and phosphorylates CLOCK. Whether the repressor complex exists and functions as a single homogeneous complex is unclear; this study does not rule out the possible existence of populations of repressor complexes varying in composition of individual PERs and CRYs. Although there are many reports showing the CLOCK–BMAL1, PER2–CRY1, CRY1–CLOCK–BMAL1 structures by NMR and x-ray diffraction (19, 38, 39), there are no structures of the complete complexes containing full-length proteins. Further work with methods such as cryo-EM may help in future investigations of circadian complexes, and the results from this study on nuclear circadian protein assemblies should facilitate these efforts.

## Experimental Procedures

### Animals

Wildtype C57BL/6J mice were purchased from Jackson Laboratory and bred in house. *Per1/2*^*−/−*^ double knockout mice in the C57BL/6J background were maintained as homozygotes and were initially generated by crossing *Per1*^*−/−*^ and *Per2*^*−/−*^ mice (40, 41). *Cry1/2*^*−/−*^ double knockout mice in the C57BL/6J background were also bred as homozygotes (42). All mice were maintained in a 12-h light:12-h dark condition for at least 14 days before collecting liver samples every 4 h over a 24-h cycle. Males and females, 12 to 24 weeks of age, were used interchangeably. All mice were handled in accordance with a protocol approved the UNC-CH Institutional Animal Care and Use Committee.

### Preparation of nuclear extracts

Over one circadian cycle, fresh livers were harvested from mice of indicated genotypes and immersed in cold PBS. After washing with PBS and mincing into small pieces the livers were washed twice with ice-cold PBS again. Tissues were then homogenized in nuclear homogenization buffer (10 mM Hepes-KOH [pH 7.6], 15 mM KCl, 0.1 mM EGTA [ethylene glycol-bis(β-aminoethyl ether)-N,N,N′,N′-tetraacetic acid] [pH 8.0], 0.5 mM EDTA [pH 8.0], 0.5 mM spermidine, 0.15 mM spermine, 0.5% Tergitol NP-10, 1 mM DTT [dithiothreitol], 1 mM PMSF (phenylmethylsulfonyl fluoride), protease inhibitors [Roche] and phosphatase inhibitors 2 and 3 [Sigma]) using 4 ml/1 g liver and using a chilled Teflon homogenizer (20 strokes) and then a 15-ml Dounce homogenizer (Kontes Glass) Pestle-A (20 strokes). The homogenate (5 ml) was mixed with 21 ml of ice-cold buffer (nuclear homogenization buffer containing 2.2 M sucrose). The sample was carefully layered on a 10-ml chilled 2 M sucrose cushion (10 mM Hepes-KOH [pH 7.6], 15 mM KCl, 2 mM EGTA [pH 8.0], 2 mM EDTA [pH 8.0], 2 M sucrose) in an ultracentrifugation tube (Beckman, 50 Ultra-clear tubes, 25 × 89 mm). Nuclei were pelleted in a SW27 rotor at 24,000 rpm, 4 °C, 90 min, and the supernatant was discarded. One milliliter of ice-cold nuclear pellet washing buffer (10 mM Hepes-KOH [pH 7.6], 100 mM KCl, 1.5 mM MgCl_2_, 0.1 mM EGTA [pH 8.0], 0.1 mM EDTA with protease inhibitors [Roche]) was added, and nuclei were incubated on ice 10 min and then gently resuspended, by using clipped pipette tips to avoid destruction of nuclei, and then transferred to a 15-ml conical tube. This process was repeated several times to collect all the pellet (total about 5–8 ml washing buffer). Nuclei were pelleted (clinical tabletop centrifuge at 2000 rpm, 5 min, 4 °C), and the pellet was resuspended again with 1 ml washing buffer, then transferred to a 1.5-ml Eppendorf tube. Nuclei were pelleted again (Benchtop Eppendorf Centrifuge, 4000 rpm, 5 min, 4 °C) and then incubated with nuclei lysis buffer (10 mM Hepes-KOH [pH 7.9], 400 mM KCl, 1.5 mM MgCl_2_, 0.1 mM EGTA [pH 8.0], 0.5 mM EDTA, 0.5% Triton X-100, protease inhibitors [Roche] and phosphatase inhibitors 2 and 3 [Sigma]) using 150 μl/50 mg pellet, for 50 to 60 min (with vortexing every 5 min) at 4 °C. Insoluble materials were removed by centrifugation (21,000*g*, 20 min, 4 °C). The supernatant served as nuclear extract for analysis by glycerol gradient centrifugation and gel filtration chromatography. Nuclear extract preparation described here is essentially the same as described (26), except in this study, nuclei were washed more extensively and high salt nuclear lysis buffer was added during the first step, rather than incrementally.

### Glycerol gradient centrifugation

Glycerol gradient centrifugation was performed as described (15, 43). Mouse liver nuclear extract (60–80 μg), obtained from one mouse sacrificed at ZT19, was mixed with reference proteins (bovine thyroid thyroglobulin [58.3 μg], sweet potato beta-amylase [14.6 μg] and chicken ovalbumin [29 μg] (44)) in a volume of 50 μl, in buffer containing 10 mM Hepes pH 7.9, 100 mM KCl, 1.5 mM MgCl_2_, 0.1 mM EDTA (pH 8.0), 0.1 mM EGTA (pH 8.0), 5% Glycerol, and 1 mM DTT. After centrifugation, the protein solution was layered on top of a 2.8-mL 10% to 30% glycerol gradient containing, in addition to glycerol, Tris (pH 8.0) 25 mM, NaCl 100 mM, and dithiothreitol 2 mM. The gradients were spun for 6 h at 54,000 rpm in an SW60 rotor at 4 °C. Fractions of approximately 240 μl were collected from the bottom of the tube, and 27-μl aliquots were analyzed by immunoblot analysis. Three gels were run for each gradient; one was stained with Coomassie blue to locate reference proteins, and the others were probed by immunoblotting to locate clock proteins. The sedimentation coefficient of known standard proteins *versus* fraction was plotted, and the linear fit to the data points was used to interpolate the sedimentation coefficients of proteins or complexes from peak elution fraction identified by blotting.

### Gel filtration chromatography

Gel filtration chromatography was performed as described previously with minor modifications (43). A Superose 6 Increase 10/300 GL (GE; 29091596) gel filtration column was equilibrated at room temperature in a buffer with 10 mM Hepes (pH 7.9), 200 mM KCl, 1.5 mM MgCl_2_, 0.1 mM EGTA, 0.1 mM EDTA, and 20% glycerol. Mouse liver nuclear extracts (4–7 μg/μl) obtained from one mouse for each different circadian time point for WT, *Per1/2*^*−/−*^, and *Cry1/2*^*−/−*^mice were diluted to 100 mM (final) KCl by addition of dilution buffer (10 mM Hepes pH 7.9, 1.5 mM MgCl2, 0.1 mM EDTA, [pH 8.0], 0.1 mM EGTA [pH 8.0], 20% glycerol). After ultracentrifugation (TSL 55; 40,000 rpm, 1 h), 400 to 600 μl samples were concentrated with Centricon Filter Units (30-kDa cutoff; Millipore), 50 to 90 μl protein solution was loaded onto the column, and the column was developed with the same buffer. The standard proteins used were Ovalbumin (Rs 3.05 nm, 45 kDa), bovine serum albumin (Rs 3.55 nm, 66.5 kDa), rabbit muscle aldolase (Rs 4.8 nm, 160 kDa), horse spleen ferritin (Rs 6.1 nm, 440 kDa), bovine thyroglobulin (Rs 8.5 nm, 669 kDa), IgM (Rs 12.1 nm, 990 kDa), and Dextran (marker for void volume). Elution profiles of the proteins of interest were determined by immunoblot analysis. A plot of peak elution volume of standard proteins *versus* their Stokes radius was constructed and a linear fit was plotted; from this standard curve the Stokes radius of circadian proteins/complexes was interpolated based upon peak elution volume of the protein/complex.

### Calculation of protein complex mass

The methodology was as described (34). The proteins of interest in the nuclear extracts were resolved with a gel filtration column calibrated with protein standards to determine the Stokes radius Rs. The extracts were also centrifuged through glycerol gradients to determine the sedimentation coefficient *S* of the target circadian proteins, again based upon comparison with standards with known S values. Mass *M* was then calculated using the formula:

M=4,205(SRs)

where *S* is in Svedberg units, *Rs* is in nanometer, and *M* is in Daltons.

### Antibodies and immunoblot analysis

For immunoblot analysis, protein samples from mouse liver nucleus/cytoplasm, IP, glycerol gradient centrifugation, and gel filtration chromatography were separated by SDS-PAGE and transferred to 0.45-μM nitrocellulose membranes (Bio-Rad, 1620115) by the Trans-Blot Turbo transfer system (Bio-Rad). The blots were blocked with 5% nonfat dry milk diluted in 1×PBST (PBS with 0.1% TWEEN-20) at least 1 h at room temperature and then incubated with primary antibodies overnight at 4 °C in 1×PBST. The primary antibodies used in this study were as follows: anti-mCRY1 (IgM) antibodies were described previously (29); anti-PER2 (Alpha Diagnostics, PER21-A), anti-CLOCK (Bethyl Laboratories, A302-618A), anti-BMAL1(Bethyl Laboratories, A302-616A), anti-CK1δ (Thermo Fisher Scientific, MA5-17243), and anti-CK1δ (Novus biological, NBP1-21376) were from commercial sources. After washes with 1×PBST, membranes were incubated with the corresponding secondary antibody for about 1 h at room temperature. The membranes were imaged using ECL (enhanced chemiluminescence) substrate (Bio-Rad, 170-5061) after washes with 1×PBST. The blot membrane was subjected to densitometric scanning.

### Immunoprecipitation of PER2/CRY1 and LC-MS/MS analysis

Mouse liver nuclear extracts obtained from three mice (for each sample) sacrificed at different circadian time points for WT, *Per1/2*^*−/−*^, and *Cry1/2*^*−/−*^mice were diluted to 150 mM (final) KCl and subjected to high-speed centrifugation as was done before application to the sizing column. For CRY1 IP, 5 ul CRY1 antibody was added to 15 ul protein L magnetic beads (Thermo Scientific, 88849) for at least 5 h, then washed with 150 mM KCl nuclei lysis buffer. Then, nuclear extract from three livers was added to the beads for overnight incubation. The beads were then washed 6 times with 150 mM nuclei lysis buffer. Bound proteins were eluted by incubation with CRY1 peptide (NSNGNGGLMGYAPGENVPSC) (29). For PER2 IP, 4 ug PER2 antibody was added to 15 ul Dynabeads protein G (Thermo Fisher Scientific, 10004D) for at least 5 h and then washed with 150 mM KCl nuclei lysis buffer. Then, mouse liver nuclear extract samples were added to the beads for overnight incubation. The beads were then washed 6 times with 150 mM nuclei lysis buffer. Bound proteins were eluted by boiling in SDS sample buffer. We conducted three independent biological replicates of each experiment.

LC-MS/MS analysis was performed as described previously with minor modifications (45). IP samples were fractionated on 10% SDS-PAGE gel, and then protein bands were tryptic digested at 37 °C for 16 h. Peptides were extracted and desalted with C18 StageTips. Desalted peptides were dissolved in 0.1% formic acid for LC-MS/MS analysis with an Easy nanoLC 1200 coupled to a Q-Exactive HFX mass spectrometer. Peptides were loaded onto a 15-cm C18 RP column (15 cm × 75 μm ID, C18, 2 μm, Acclaim Pepmap RSLC, Thermo Fisher) and eluted with a gradient of 5 to 30% buffer B (80% acetonitrile in 0.1% formic acid) at a constant flow rate of 300 nl/min for 17 min followed by 30% to 40% B for 3 min and 100% B for 10 min. The Q-Exactive HFX was operated in the positive-ion mode with a data-dependent automatic switch between survey Full-MS scan (*m/z* 350–1400) and higher energy collision dissociation mass spectrometry acquisition of the top 15 most intense ions. Survey scans were acquired at a resolution of 60,000 at *m/z* 200. Up to the top 15 most abundant isotope patterns with charge ≥ 2 from the survey scan were selected with an isolation window of 1.4 *m/z* and fragmented by higher energy collision dissociation with normalized collision energies of 27. The maximum ion injection time for the survey scan and the MS/MS scans was 100 ms, and the ion target values were set to 1e5 and 1e4, respectively. Selected sequenced ions were dynamically excluded for 20 s. There were three biological replicates, and each sample was subjected to two technical LC-MS/MS replicates.

Mass spectra processing and peptide identification was performed using the MaxQuant software version 1.6.10.43 (Max Planck Institute). All peptide matching searches were performed against the UniProt *Mus musculus* protein sequence database (UP000000589). A false discovery rate for both peptide-spectrum match and protein assignment was set at 1%. Search parameters included up to two missed cleavages at Lys/Arg on the sequence, oxidation of methionine, and protein N-terminal acetylation as a dynamic modification. Carbamidomethylation of cysteine residues was considered as a static modification. Data processing and statistical analysis were performed on Perseus (Version 1.6.10.50). Label-free quantification was performed on biological and technical replicate runs, and a two-sample *t* test statistic was used to report statistically significant fold changes (false discovery rate = 0.05, fold change >2).

## Data availability

All data are contained within the article except for the spectrometry proteomics data, which have been deposited to the ProteomeXchange Consortium *via* the PRIDE partner repository with the dataset identifier PXD037841.

## Supporting information

This article contains supporting information.

## Conflict of interest

The authors declare that they have no conflicts of interest with the contents of this article.
