# Supplementary material for: Analysis of mammalian circadian clock protein complexes over a circadian cycle
Source: J Biol Chem. 2023 Jan 20;299(3):102929. doi: 10.1016/j.jbc.2023.102929 (PMC9950529; doi:10.1016/j.jbc.2023.102929)
Supplement: Supporting information [file mmc3.docx]

**Analysis of Mammalian Circadian Clock Protein Complexes Over a Circadian Cycle**

Xuemei Cao#, Li Wang#, Christopher P. Selby, Laura A. Lindsey-Boltz, Aziz Sancar*

Department of Biochemistry and Biophysics, University of North Carolina School of Medicine, Chapel Hill, North Carolina 27599-7260

# Equal contribution

*For correspondence: [aziz_sancar@med.unc.edu](mailto:aziz_sancar@med.unc.edu)

**Supplementary Figures**

**
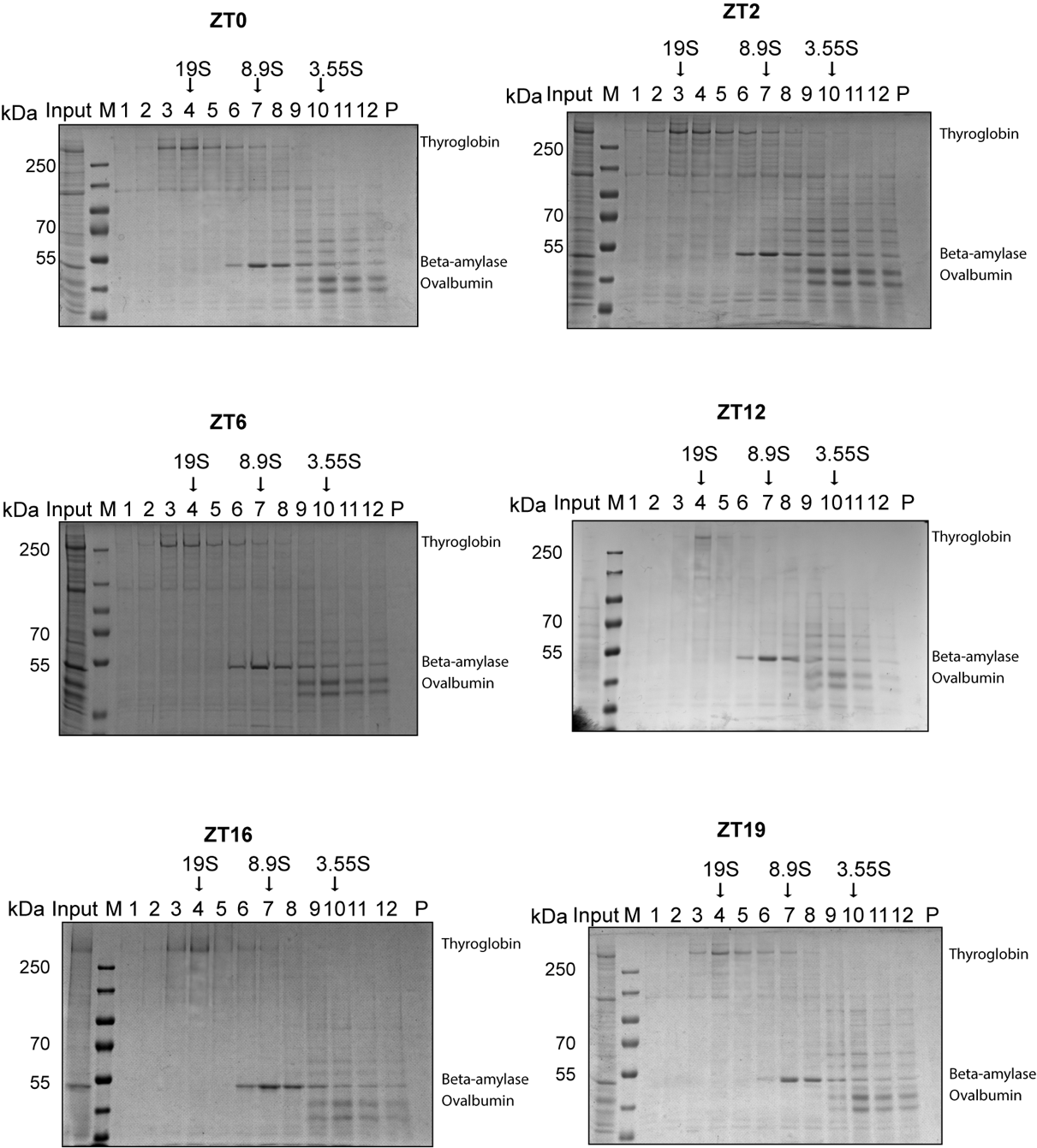
**

**Figure S1. Mobility of protein standards in glycerol gradients.** Gels were loaded with the same samples analyzed in Figure 1 and stained with Coomassie blue to determine peak elution fractions for the protein standards thyroglobulin, beta-amylase and ovalbumin.


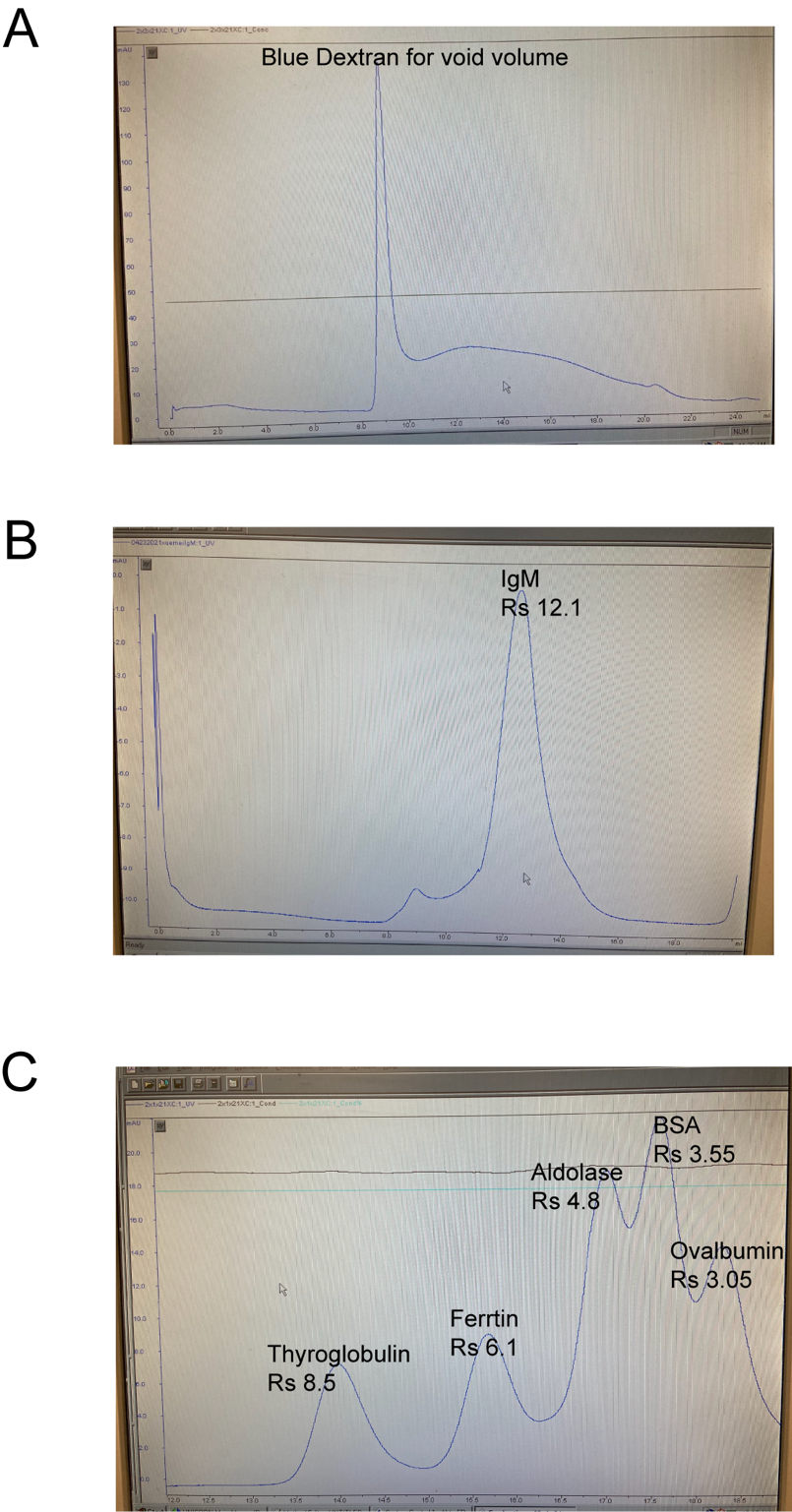


**Figure S2. Standardization of sizing column.** Standards blue dextran (**A**), IgM (**B**), thyroglobulin, ferritin, aldolase, BSA and ovalbumin **(C**) were resolved on the Superose 6 Increase 10/300GL column to determine peak elution fractions. Proteins were detected by UV absorbance, and scans of the elution profiles are shown.


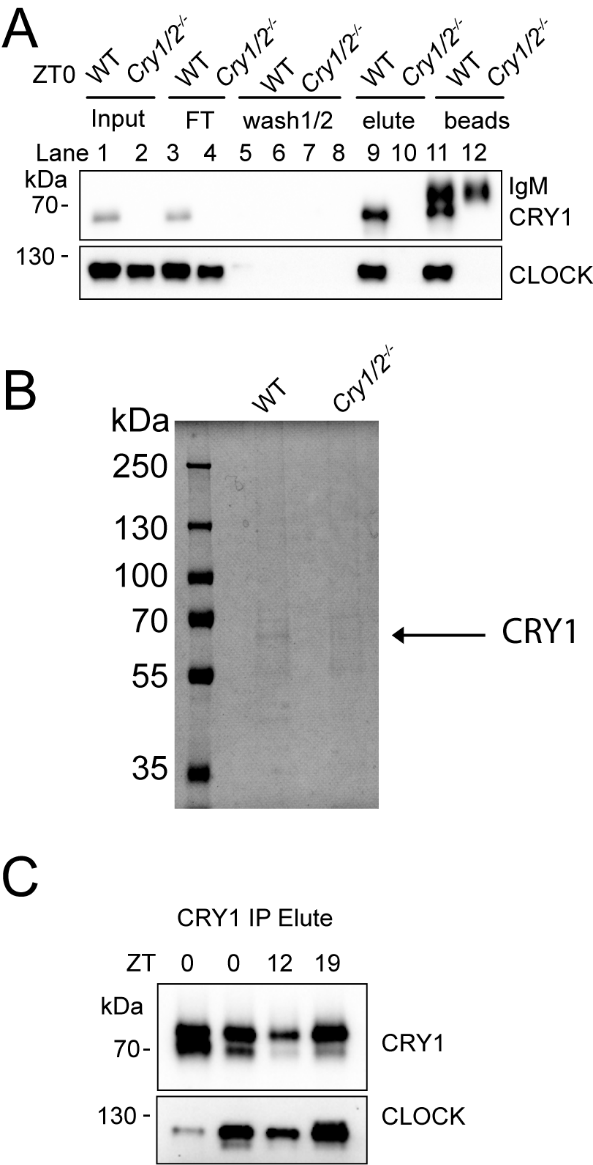


**Figure S3. Purification of CRY1 complexes from mouse liver nuclear extracts.** Negative controls for precipitation of CRY1 from wild-type and *Cry1/2^-/-^* double knockout mouse liver extracts with anti-CRY1 antibodies. **(A)** Immunoblot showing CRY1 purification from ZT0 extract with anti-CRY1 antibodies. FT is flow-through, that is, the supernatant after binding extract to the sample and pelleting. In this case, a significant amount of CRY1 did not bind to the resin. **(B)** CRY1 purified from ZT0 mouse nuclei at ZT0 was analyzed by SDS gel electrophoresis and stained with Coomassie blue. **(C)** Immunoblots showing CRY1 purified from ZT0, ZT12, and ZT19 mouse nuclei.


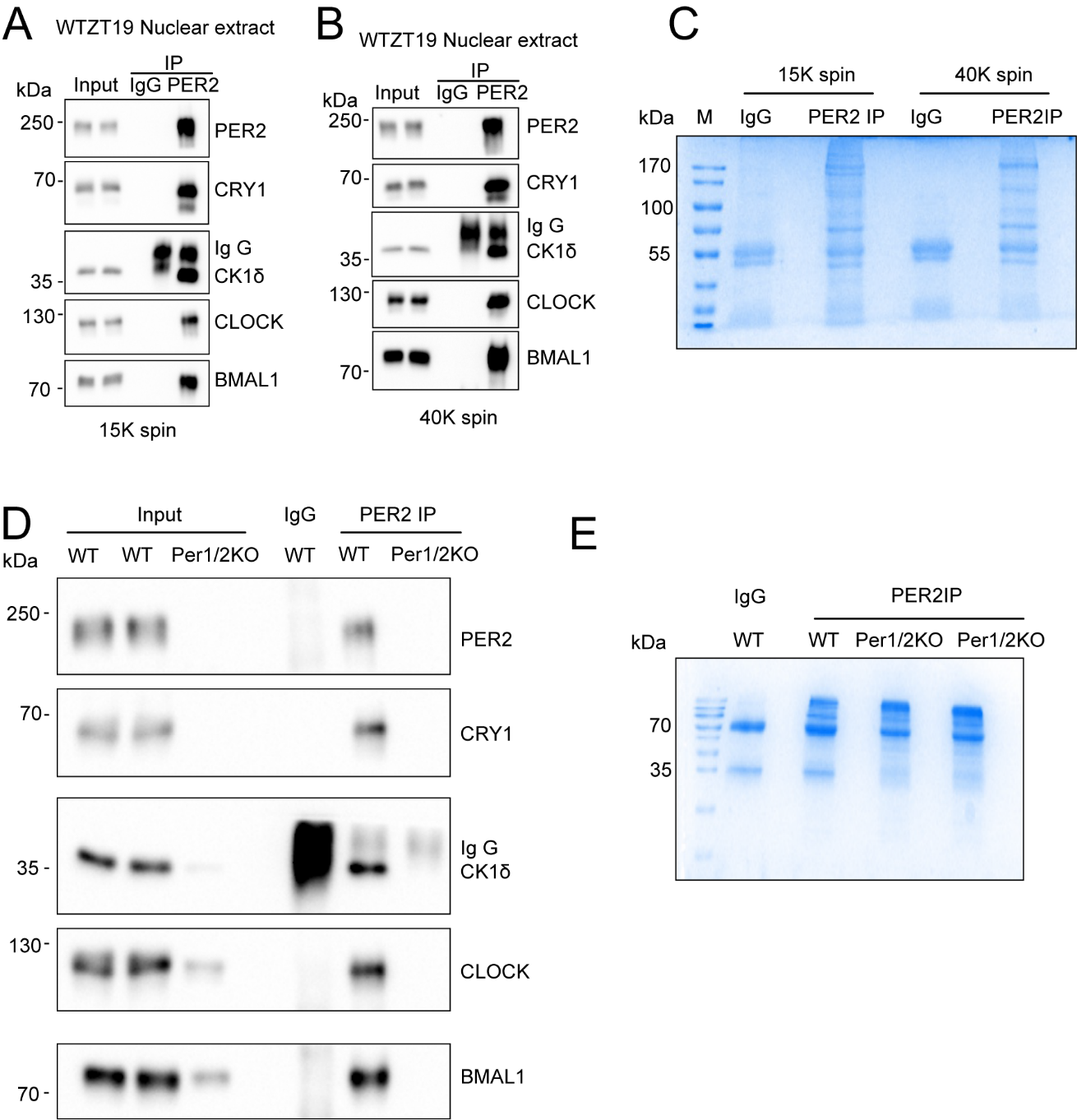


**Figure S4. Purification of PER2 complexes from mouse liver nuclear extracts.** Nuclear extracts from ZT19 were centrifuged at either 15K low **(A)** or 40K high **(B)** speeds before immunopurification and analysis by western blotting. Similar patterns of input proteins and elution of clock proteins with PER2 antibodies but not IgG control show that PER2 complexes were isolated from both extracts. Purified proteins, stained with Coomassie blue, are shown in **(C).** Extracts were prepared using high speed or the more commonly used low speed centrifugation. We used high speed centrifugation since preliminary results showed many more interactions when low speed extracts were used for immunoprecipitation. (High speed extracts were also used for gel filtration chromatography since the low-speed extracts did not properly enter the column.) **(D)** shows the comparable purification of clock proteins from ZT19 high-speed nuclear extracts from wild-type (WT) or *Per1/2^-/-^* double knockout mice as a negative control. **(E)** shows a Coomassie blue stained gel with the purified samples. Repeats for the *Per1/2*^-/-^ nuclear extract (KO) are shown.
